# Thylakoid-Deposited Micro-Pillar Electrodes for Enhanced Direct Extraction of Photosynthetic Electrons

**DOI:** 10.3390/nano8040189

**Published:** 2018-03-25

**Authors:** DongHyun Ryu, Yong Jae Kim, Seon Il Kim, Hyeonaug Hong, Hyun S. Ahn, Kyunghoon Kim, WonHyoung Ryu

**Affiliations:** 1 Department of Mechanical Engineering, Yonsei University, Seoul 03722, Korea; m_school@naver.com (D.R.); blackeva8@gmail.com (Y.J.K.); seonilkim81@gmail.com (S.I.K.); hyeonaug@gmail.com (H.H.); 2 Department of Chemistry, Yonsei University, Seoul 03722, Korea; ahnhs@yonsei.ac.kr; 3 School of Mechanical Engineering, Sungkyunkwan University, Suwon 16419, Korea

**Keywords:** photosynthesis, micro-pillar anodes, photosynthetic fuel cells, thylakoids, solar energy conversion

## Abstract

Photosynthesis converts solar energy to electricity in a highly efficient manner. Since only water is needed as fuel for energy conversion, this highly efficient energy conversion process has been rigorously investigated. In particular, photosynthetic apparatus, such as photosystem II (PSII), photosystem I (PSI), or thylakoids, have been isolated from various plants to construct bio-hybrid anodes. Although PSII or PSI decorated anodes have shown potentials, there still remain challenges, such as poor stability of PSII-based systems or need for electron donors other than water molecules of PSI-based systems. Thylakoid membranes are relatively stable after isolation and they contain all the necessary photosynthetic apparatus including the PSII and PSI. To increase electrical connections between thylakoids and anodes, nanomaterials such as carbon nanotubes, nanowires, nanoparticles, or graphene have been employed. However, since they rely on the secondary electrical connections between thylakoids and anodes; it is desired to achieve larger direct contacts between them. Here, we aimed to develop micro-pillar (MP) array anodes to maximize direct contact with thylakoids. The thylakoid morphology was analyzed and the MP array was designed to maximize direct contact with thylakoids. The performance of MP anodes and a photosynthetic fuel cell based on MP electrodes was demonstrated and analyzed.

## 1. Introduction

**Figure 1:**
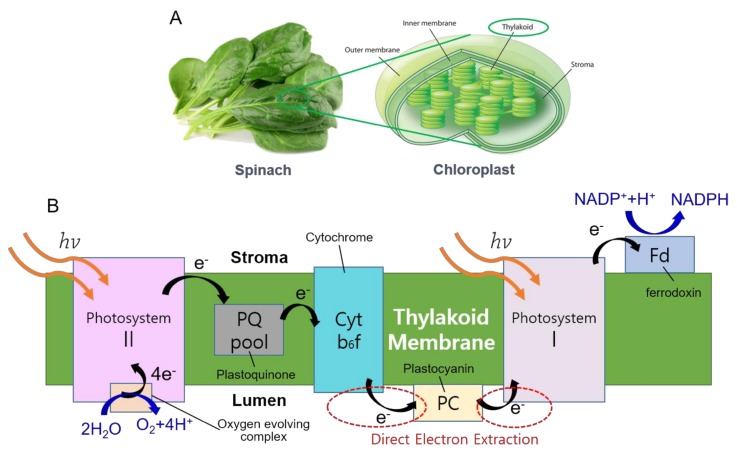
(**a**) Schematic of thylakoid membranes and chloroplasts from spinach leaves; and (**b**) photosynthetic electron pathway in thylakoid membrane.

Plant photosynthesis generates photosynthetic electrons (PEs) by splitting water into protons, oxygen, and electrons with the aid of solar energy. Solar photons energize the PEs and excite them to a higher energy level, and PEs are transferred through various photosynthetic apparatus in thylakoid membranes with a series of redox reactions (Figure 1). There have been many attempts to directly or indirectly extract PEs from microbial and photosynthetic bacteria [1,2]. Photosystem II (PSII) have been isolated from plant cells and experimented to assess the feasibility as a stand-alone molecular complex that can continuously split water molecules and generated PEs [3,4,5]. Photosystem I (PSI)-based systems demonstrated stable and enhanced performance as bio-solar energy systems with various anode materials [6,7,8]. However, the poor stability of the isolated PSII complexes or need for additional electron donors other than water for PSI-based systems still remain as challenges for their further development. More recently, direct extraction of PEs from living algal cells by nanoelectrode insertion was also demonstrated with high efficiency and long-term stability [9,10]. However, the difficulty of scale-up of this approach also needs to be further investigated.

Thylakoids contain all the photosynthetic apparatus, such as PSII, a plastoquinone pool, cytochrome b_6_f complex, plastocyanin, and PSI. They are relatively easy to be isolated from plant cells and remain stable after isolation compared to other isolated photosynthetic protein complexes. With these advantages, there have been many investigations that utilized thylakoids for bio-solar energy conversion. A key challenge of thylakoid-based bio-solar energy conversion is maximizing electrical connections between thylakoids and main anodes of a PE harvesting system. With this regard, many nanomaterials, including carbon nanotubes (CNTs) [11,12], nanowires [13], and two-dimensional materials, such as graphene [14,15], were incorporated with isolated thylakoids, and demonstrated their enhanced performance compared to systems without such nanomaterials. However, since these approaches rely on secondary connections by the nanomaterials between thylakoids and an anode, a way to increase direct contact between thylakoids and an anode may further enhance the performance of PE harvesting.

In this work, we propose a novel design of an anode to accommodate thylakoids more efficiently and maximize direct contact between thylakoids and the anode surface. Micro-pillar (MP)-shaped electrodes were designed and fabricated based on the morphology of isolated thylakoids. It was hypothesized that three-dimensional MP electrodes could accommodate thylakoids between the micro-pillars such that their direct contact area could be increased compared to that of flat electrodes. Thylakoids isolated from spinach leaves were carefully analyzed to obtain their morphology and dimensions. Based on the thylakoid geometry, the optimum dimension of MP electrodes (diameters and spacing between MPs) was determined. MP electrodes were fabricated using metal-assisted chemical (MAC) etching. The controlled amount of thylakoids was drop cast on both flat and micro-pillar electrodes. Photosynthetic (PS) currents from the thylakoid-cast MP electrodes were analyzed under various conditions. Finally, a prototype of a PS bio-solar fuel cell was prepared and their performance was further studied.

## 2. Materials and Methods 

### 2.1. Materials

4-(2-Hydroxyethyl)piperazine-1-ethanesulfonic acid (HEPES, H3375), sodium chloride (NaCl, S7653), magnesium chloride (MgCl_2_, M8266), D-sorbitol (S3889) were purchased from Sigma-Aldrich (St. Louis, MO, USA) for isolation of thylakoids. Hydrogen peroxide (H_2_O_2_, HDE0-18005) and hydrogen fluoride (HF, HDC0-00101) were purchased from Duksan (Cheonan, Korea), gold etchant (standard, 651818) was purchased from Sigma-Aldrich for metal-assisted chemical (MAC) etching. Nafion 212 and carbon paper were purchased from the Nanoholdings Ltd. (Seoul, Korea). Potassium ferricyanide (K_3_Fe(CN)_6_, 702587) and potassium nitrate (KNO_3_, P8394) as redox mediators, were purchased from Sigma-Aldrich. All aqueous solutions were prepared by using ultrapure de-ionized water (>18 MΩ·cm^−1^). 

### 2.2. Thylakoids Isolation

Thylakoids were isolated from spinach by following the protocols as described previously [16]. Spinach was purchased from a local market and washed with tap water first and then distilled water. After removing stems from the spinach, the leaves were cut into small pieces with scissors. They were ground in a phosphate buffer solution (pH 7, 50 mM sodium phosphate, 10 mM NaCl, 5 mM MgCl_2_, 300 mM D-sorbitol) using a laboratory blender (Cole-Parmer, 8011EG, Waring) at a speed of 22,000 rpm for 20 s. The ground spinach leaves were filtered through a 20 μm nylon mesh and centrifuged at 3000× *g* for 10 min. After the supernatants were removed, the pellets were re-suspended with a 5 mM MgCl_2_ solution and gently pipetted for 10 s to induce osmotic shock to rupture the chloroplast membranes. A wash medium (pH 7, 50 mM sodium phosphate, 10 mM NaCl, 5 mM MgCl_2_, 300 mM D-sorbitol) was added to the solution and centrifuged at 4000× *g* for 5 min. The isolated thylakoid membranes were suspended in the wash medium and stored in a refrigerator at 4 °C before use. All centrifugation processes were conducted at 4 °C.

### 2.3. Measurement of Chlorophyll Concentration

The concentration of the chlorophyll inside the isolated thylakoids was spectroscopically determined as described previously [17]. Four microliters of isolated thylakoids were suspended in 2 mL of 80% (*v*/*v*) acetone and the suspended solution was gently pipetted, and the absorbance of the solution was measured at 645 nm and 663 nm. Using the conversion equation by the previous work, the chlorophyll concentration in the isolated thylakoids was determined as 4 to 9 mg·chl mL^−1^.

### 2.4. SEM Imaging of Thylakoids

Isolated thylakoids were diluted with distilled water to image the precise morphology of single or multiple vesicles. A diluted thylakoid solution was dropped on a silicon wafer piece whose surface was treated with oxygen plasma to make the surface hydrophilic for thin and uniform spreading of thylakoids on the substrate. The thylakoid-deposited substrate was dried in a convection oven at 36 °C for two days. A few nanometer thick Pt layer was coated before SEM imaging (JSM-7001F, JEOL Ltd., Akishima, Japan).

### 2.5. Fabrication of MP Electrode

**Figure 2:**
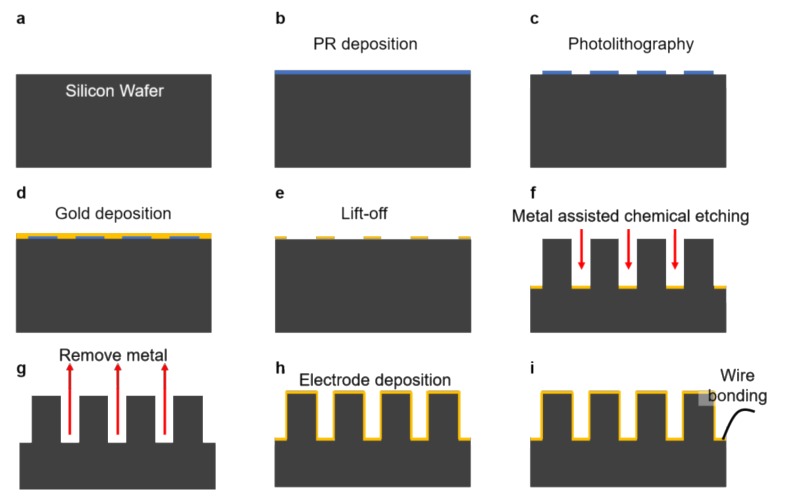
Fabrication process of micro-pillar (MP) electrode based on metal-assisted chemical (MAC) etching.

MP electrodes were fabricated by the MAC etching process (Figure 2). First, photoresist (PR) (TDUR-P902, TOKYO OHKA KOGYO CO., Ltd., Kawasaki, Japan) was coated on a silicon wafer using a spin coater (Figure 2b). Photolithography was conducted to obtain a micro circle array pattern (Figure 2c) using the KrF stepper (PAS5500, ASML, Veldhoven, The Netherlands). An E-beam evaporator (UEE, ULTECH CO., Ltd., Daegu, Korea) was used to deposit a Ti adhesion layer (2 nm thick) and a Au catalyst layer (20 nm thick) at a deposition rate of 0.1 nm/sec (Figure 2d). After the metal deposition, a lift-off process was conducted to obtain a desired pattern of Au catalyst on the silicon wafer (Figure 2e). MAC etching was conducted using a mixed solution comprised of 12.5% (*v*/*v*) HF, and 1.5% (*v/v*) H_2_O_2_ (Figure 2f). A gold etchant was used to remove the Au catalyst layer (Figure 2g). Finally, a Au layer (50 nm thick) was deposited over the MP structures using a RF magnetron sputter (KOVA, Daegu, Korea) (Figure 2h). The metalized MP electrodes were wire-bonded using silver paste and insulated with epoxy glue (Figure 2i).

### 2.6. Measurement of Photosynthetic Currents

Electrochemical measurement was conducted in a three-electrode setup comprised of MP electrode as a working electrode, Ag/AgCl as a reference electrode, and a Pt mesh as a counter electrode based on electrochemical analyzsis (CompactStat, Ivium, Eindhoven, The Netherlands). The entire measurement setup was placed in a custom-built Faraday cage with a built-in halogen lamp (LG-PS2, Olympus, Shinjuku, Japan) that was controlled from the outside. Unless mentioned otherwise, a bias potential of 0.4 V was applied between an MP anode and Pt mesh counter electrode. In a standard measurement, light intensity was 10 mW cm^−2^. The amount of thylakoid coating was maintained at a value of 2.96 mg·chl/mL. PCs were measured in a manner of chronoamperometry (CA) to monitor light-triggered PC values, while cyclic voltammetry (CV) was performed to identify which photosynthetic apparatus contribute to the light-triggered PC generation.

## 3. Results

### 3.1. Design and Fabrication of MP Electrodes

**Figure 3:**
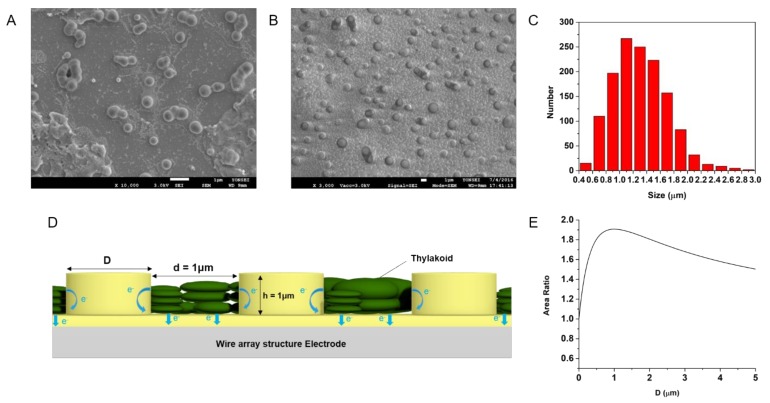
SEM images of (**A**) thylakoids that formed multiple vesicles and grana stack; and (**B**) single vesicle thylakoids by diluted isolation; (**C**) Histogram of thylakoid size distribution; (**D**) Schematic design of pillar-structured electrode; (**E**) Plot of the area ratio of an electrode surface with 1 μm gap for varying pillar diameter D compared to a flat electrode.

After isolation, there were individual thylakoid vesicles or multiple stacks of thylakoid grana as shown in Figure 3a. When thylakoid solutions were further diluted, more accurate size distribution of individual thylakoids could be estimated (Figure 3b). The size distribution of thylakoids (*n* = 1363) was analyzed by a histogram as shown in Figure 3c. The average diameter of thylakoids was 1.3 μm with the range from 400 nm to 3 μm. The size distribution matches well with other previous reports [18,19,20]. In order to maximize the surface area of an MP electrode, the diameter of each pillar was optimized for the given size of thylakoids. Considering the design shown in the inset of Figure 3d, the area could be estimated as follows:

(1) $$\mathrm{Surface}\;\mathrm{area}=\;\frac{2}{\sqrt{3}}\frac{10^{8}}{{\left(D+d\right)}^{2}}\times \left(\frac{\sqrt{3}}{2}{\left(D+d\right)}^{2}+\pi Dh\right)$$

where *D* is the diameter of each pillar, *d* is the distance between pillars, and h is the height of each pillar. Since the average diameter of thylakoids was about 1.3 μm, the spacing between pillars (*d*) can be set to be 1 μm such that each thylakoid could fit tightly within the spacing between pillars. With this condition, the surface area of an MP electrode becomes the largest when *D* is 1 μm from the Equation (1). Figure 3e shows how the area ratio of the electrode surface changes for change of *D*. Thus, pillar diameter (*D*) was set to be 1 μm for this study.

**Figure 4:**
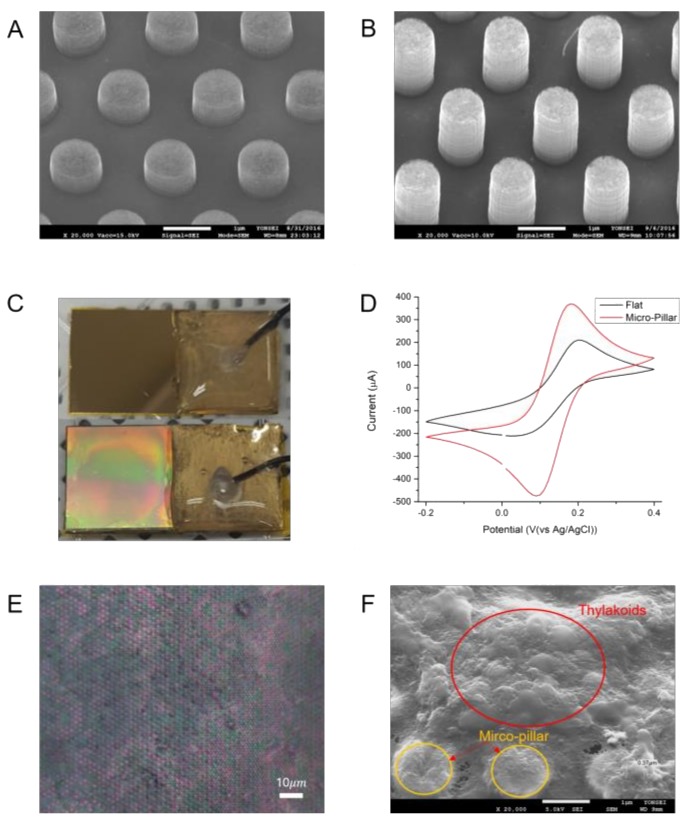
SEM image of MP electrodes with aspect ratio of (**A**) 1.0 (MACE1) and (**B**) 1.5 (MACE2); (**C**) Au-coated flat (above) and MP (below) electrodes; (**D**) Cyclic voltammetry to determine the electrochemical surface of a flat and MP electrode using a solution of 10 mM potassium ferricyanide and 100 mM potassium nitrate. The potential range was −0.2 V to 0.4 V and the scan rate was 20 mV/s; (**E**) Optical microscope image of thylakoids on MP electrodes; (**F**) SEM image of thylakoids on MP electrodes.

Based on the MP electrode design, MP electrodes with aspect ratios of 1 (MACE 1) and 1.5 (MACE 2) were fabricated in a fashion of a hexagonal array by MAC etching as shown in Figure 4a,b. Since the etched depth is proportional to the etching time, the aspect ratios of those MACE 1 and 2 were determined by their etching times. The etching time for MACE 1 was 10 min, while the etching time for MACE 2 was 15 min. The walls of the MP electrodes were straight compared to silicon structures etched by a conventional wet etching method. Figure 4c shows the different optical reflections from a Au-coated flat silicon substrate and Au-coated MP electrodes. Uniform optical reflectance from the MP electrodes also indicates uniformly-structured MP arrays by the MAC etching process.

### 3.2. Electrochemical Surface Area of MP Electrode

The major benefit of MP electrodes is the increased surface area that participates in the electrochemical oxidation of thylakoids. It is important to understand how much enhancement in the electrochemical surface area (ECSA) of an MP electrode was achieved. Thus, CV was used to measure the ECSA of an MP electrode compared to a flat electrode (Figure 4d). Both types of the electrodes had the projected size of 1 × 1 cm^2^. CV was conducted in a standard redox solution of 10 mM potassium ferricyanide and 100 mM potassium nitrate. The potential bias range was set between −0.2 V and 0.4 V, while the scan rate was 20 mV/s. The Randles-Sevcik equation was used to compare the ECSA of an electrode like the following [21]:

(2) $$i_{p}=\left(2.69\times 10^{5}\right)n^{1.5}D^{0.5}CAv^{0.5}$$

where *i_p_* is the peak current of oxidizing reaction, *n* is the number of electrons for reaction (*n* = 1), *D* is the diffusion coefficient, *C* is the molar concentration of potassium ferricyanide, *A* is the electrochemical surface area of electrode, and *v* is the scan rate of cyclic voltammetry. 

According to the Equation (2), the peak current is proportional to the ECSA of the electrode. Since all other variables were maintained identical for both types of electrodes, the ECSA ratio of the two electrodes (ECSA of MP/ECSA of flat electrodes) can be calculated by comparing their peak currents. The peak current of a flat electrode was 190 μA, while the peak current from an MP electrode was 386 μA. That is, the ECSA of an MP electrode was 2.03 times larger than that of a flat electrode. The ECSA ratio value matches well with the ratio of the geometric surface area of the MP electrode over the flat electrode, which was 2.36. This high correlation between the ECSA and surface area values indicates about 86% of the MP electrode surface is electrochemically active and can participate in the harvesting of PEs.

### 3.3. Electrochemical Impedance Spectroscopy

**Figure 5:**
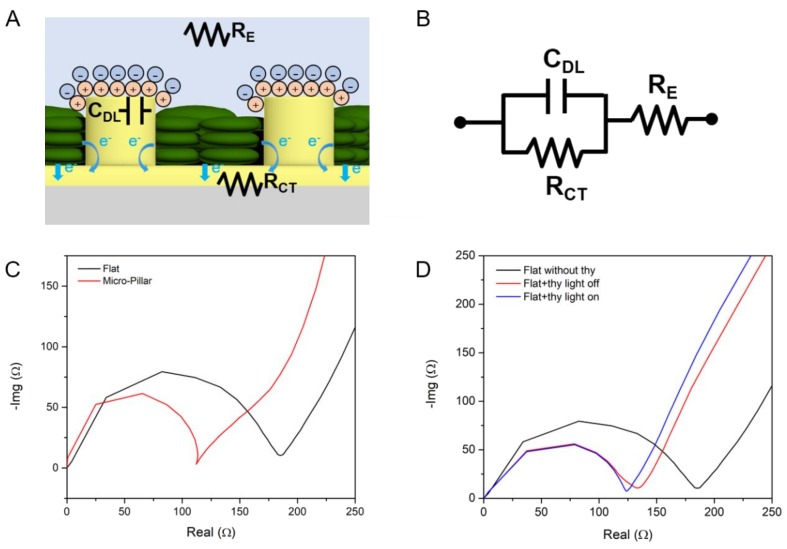
(**A**) Scheme of thylakoids-deposited MP electrode for an equivalent circuit model; (**B**) Randles circuit equivalent to the photosynthetic fuel cell anode; (**C**) Nyquist plots of a flat vs. MP electrode; (**D**) Nyquist plot of a flat electrode without thylakoids (black), with thylakoid with light off (red), with thylakoid with light on (blue).

Electrochemical impedance spectroscopy (EIS) can be used to analyze the characteristics of biological fuel cells such as microbial fuel cells and photosynthetic fuel cells [22]. Using the EIS technique, more detailed contribution of MP electrodes to the enhancement of PS currents was investigated. An equivalent electrical circuit model of thylakoid-coated MP electrodes in an electrolyte solution is shown in Figure 5a. In the model, an electrolyte solution, which is a phosphate buffer solution, can be modeled as electrolyte resistance (*R_E_*). There is an electrical double layer formed at the interface between the electrolyte and the MP electrodes. This electrical double layer functions as a capacitance (*C_DL_*). Charge transfer from thylakoids and electrolytes to the MP anode can be represented as charge transfer resistance (*R_CT_*), which is parallel to the *C_DL_*. This electrical circuit model corresponds to the Randles circuit (Figure 5b). EIS was performed using the MP anode as a working electrode with a 0.4 V bias voltage. Ag/AgCl and a Pt mesh were used as a reference electrode and a counter electrode, respectively. The applied frequency was from 10 MHz to 1 Hz in a form of AC signals with a 10 mV amplitude.

The impedance measurements of the MP vs. flat electrodes without thylakoids were compared in a Nyquist plot as shown in Figure 5c. By using the curve fitting of the EIS results (IviumSoft, Ivium Technologies B.V., Eindhoven, the Netherlands), the values of *R_E_*, *R_CT_* and *C_DL_* were estimated as shown in Table 1. Internal resistance (*R_INT_*) is the sum of *R_E_* and *R_CT_*. The charge transfer resistance of MP electrodes (109.9 Ω) was lower than the resistance of flat electrodes (165.8 Ω). This enhancement of charge transfer resistance of MP over flat electrodes is likely due to the larger ECSA of the flat electrodes. This is also supported by the fact that the ratio of the charge transfer resistance of MP over flat electrodes is 0.66, while the reciprocal value of the ECSA ratio of MP over flat electrodes is 0.49.

In order to understand the effect of thylakoids on the electrochemical performance of the electrodes, the EIS of a thylakoid-deposited flat electrode was also compared to another flat electrode without thylakoid, as shown in Figure 5d. The thylakoid concentration was 9 mg·chl/mL and a 50 μL aliquot of the thylakoid solution was dropped on a flat electrode, followed by drying for 24 h in a convection oven. As summarized in Table 2, deposition of thylakoid on a flat electrode reduced the charge transfer resistance markedly from 165.8 to 117 Ω. It is likely that the coated thylakoid created larger surface area for electrochemical reactions compared to a bare flat electrode. Illumination over the thylakoid-coated electrode reduced the resistance further down to 112.7 Ω. This additional light-assisted reduction of charger transfer resistance is ascribed to the generation of photosynthetic electrons. 

### 3.4. Measurement of Photosynthetic Currents

**Figure 6:**
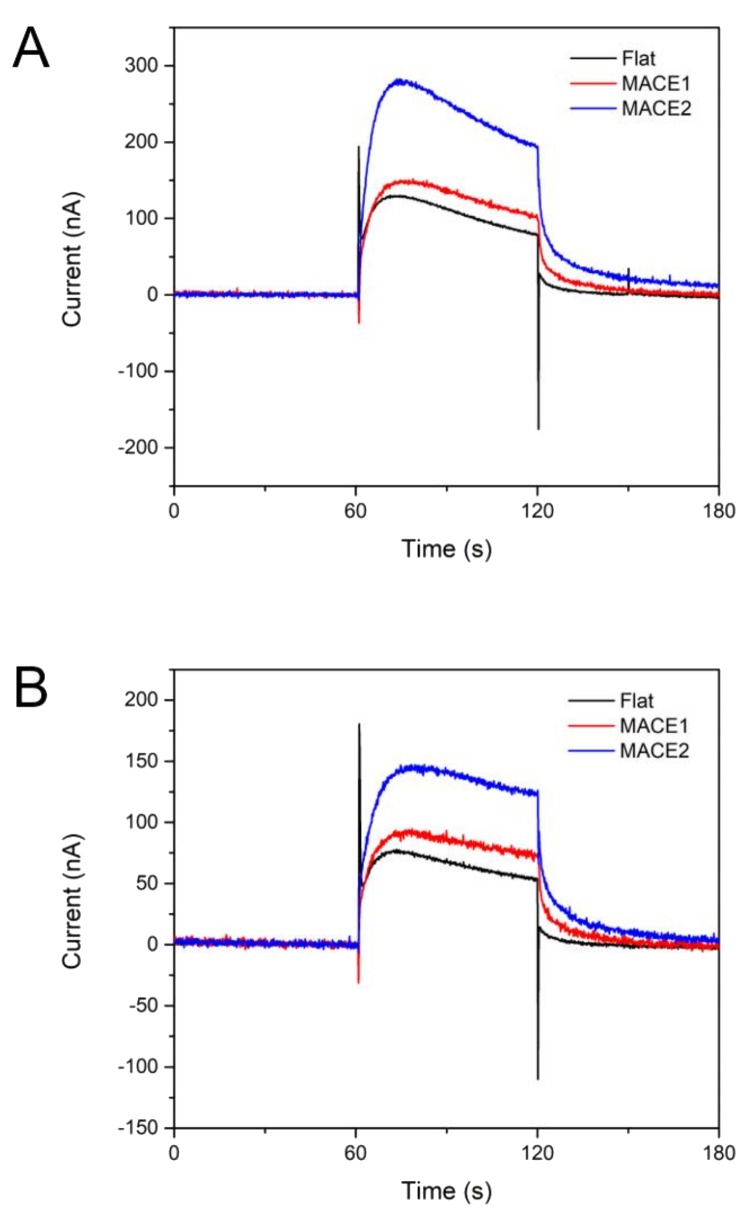
Photosynthetic currents from thylakoid-deposited flat, MACE1, and MACE2 electrodes during the (**A**) first and (**B**) second cycle of illumination. Each cycle lasted for 60 s.

PCs were measured as described in the Section 2.6. A flat electrode and two MP electrodes were prepared, and the same amount of thylakoid solution was dropped on the surface of each electrode. The light intensity was set to 10 mW/cm^2^ and 0.4V (vs. Ag/AgCl) bias voltage was applied. Light was illuminated for 60 s twice with 5 min intervals. As shown in Figure 6a, the PCs showed typical current peaks and slight decrease during each illumination cycle. The magnitudes of PCs depended strongly on the geometry of the electrodes. As anticipated, the MP electrode with higher aspect ratio (*A*/*R*) (MACE 2) resulted in the largest PCs, while the flat electrode had the smallest PCs. Compared to the flat electrode, 241% of PC was achieved in the MACE 2. Since the surface area of the MACE 2 electrode was about 236% of the flat electrode (Table 3), most of the PC enhancement is considered to originate from the increased surface area of the MACE 2 electrode. For the second illumination cycle, the magnitude of PCs decreased to 55–64% of the PC magnitude of the first cycle (Figure 6b and Table 3). 

### 3.5. Photosynthetic Fuel Cell Operation

**Figure 7:**
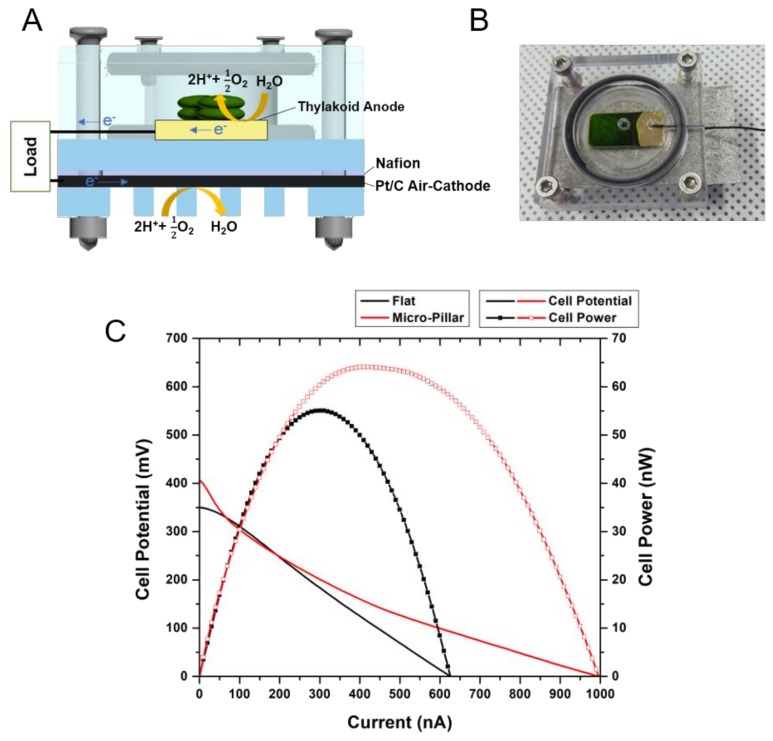
(**A**) Schematic design of air-cathode photosynthetic fuel cell; (**B**) Photograph of the fabricated photosynthetic fuel cell with thylakoid-deposited anode (green colored zone); (**C**) I-V curves of photosynthetic fuel cell with different anodes (flat electrode: black line, micro-pillar electrode: red line).

Using the thylakoid-deposited MP electrode, a photosynthetic fuel cell (PFC) was constructed and its performance was evaluated. The PFC was configured in a similar manner to a proton exchange membrane fuel cell (PEMFC) [23]. A single chamber PFC made of acryl was designed and fabricated (Figure 7a). The anodic chamber is configured to have a thylakoid-deposited MP electrode, while Pt-coated carbon (Pt/C) mesh was employed as a cathode that can exchange air between the chamber and the outside. The Pt/C cathode was used because of its superior oxygen reduction capability compared to other materials such as glassy carbon or bio-catalysts. Nafion^®^ membrane was inserted between the cathode chamber and the air-breathing Pt/C cathode. Figure 7b shows the photograph of a fabricated PFC with a thylakoid-deposited electrode (green color).

For the performance comparison of a flat and MP electrode, an I-V curve was plotted and analyzed (Figure 7c). A 20 μL aliquot of a thylakoid solution (thylakoid concentration = 9 mg·chl/mL) was dropped on the electrodes and dried for 40 h in a convection oven before use. The light intensity was 30 mW/cm^2^. The open circuit voltage (OCV) of the flat electrode was 350 mV, while the OCV of the MP electrode was 407 mV. The OCV of the flat and MP electrode cells is comparable to the value reported in a previous work [11]. The maximum power of the MP electrode cell was 64 nW, which is 17% higher than that of the flat electrode (55 nW). The maximum current of the MP electrode cell was 990 nA, which is 60% higher than that of the flat electrode cell (620 nA). It is noteworthy that the Ohmic loss of the MP cell was smaller than that of the flat cell. Such enhancement is likely because of the reduced charge transfer resistance of the MP electrode as analyzed in Section 3.3. 

## 4. Discussion

The dimensions of MP electrodes were determined based on the size of isolated thylakoids. The thylakoids isolated from spinach leave had the average size of 1 μm (Figure 3). Small features are typically employed to increase the surface-to-volume ratio of an electrode, therefore, nanoscale features are often used in many applications. However, when the size of a target object (thylakoids in our study) is not small enough compared to the feature size of an electrode, more careful design of the electrode feature is necessary. Since we aim to maximize the direct contact area between thylakoids and an electrode, use of very thin nanowire-like structure may not be the best choice for our purpose. In fact, when the area ratio of an electrode surface over flat electrode was calculated for varying dimensions of the pillar or wire structures, the maximum area ratio was predicted for the pillar diameter of 1 μm (Figure 3d inset). With micro-pillars 1 μm in diameter, the surface area of an MP array electrode is about 1.9 times larger than that of a flat electrode. A similar amount of enhancement in PC collection was observed in Figure 6. 

Fabrication of such MP array electrodes with 1 μm features is non-trivial. There have been various fabrication methods that can create nanostructures, such as nanowires smaller 100 nm or MPs larger than a few micrometers. However, creation of MPs with a 1 μm diameter with a precise spacing of 1 μm between each MP requires fabrication approaches different from well-established bottom-up vapor-liquid-solid (VLS) methods or top-down deep reactive ion etching (DRIE) techniques. MAC-etching was recently introduced to the field as a simple, but robust, fabrication method that can create highly-dense array of nanoscale to a few micrometer features with high *A*/*R* [24,25,26]. As shown in Figure 2, the photolithographically-patterned metal layer enabled precise placement of MPs with high *A*/*R*. Unlike DRIE-created structures, the side walls of MPs by MAC-etching have smooth surfaces (Figure 4a,b) and the fabricated MPs are highly uniform over an entire substrate (Figure 4c). Although MPs with A/R of up to 1.5 were fabricated in this study, higher *A*/*R* above 10 can be easily achieved by increasing etching time.

Use of electrochemical mediators facilitates electron transport between thylakoids and the anodes of photosynthetic fuel cells. Typically, this leads to extraction of much larger amount of photosynthetic electrons than direct electron extraction without mediators. However, the use of mediators is less desired due to its environmental toxicity and degrading performance over time. The degrading performance of mediators can cause the voltage loss of the systems and their instability under light and in high temperatures [27,28]. As another approach, linker molecules or electrically-conducting nanomaterials were employed to immobilize or connect thylakoids and metal electrodes. Thylakoids were immobilized on glassy carbon electrodes using carboxyphenyl groups [28], multi-wall carbon nanotubes (MWCNT) using a tethering molecule [11], 1-pyrenebutanoic acid succinimidyl ester (PBSE) or thylakoids were electrically-wired with osmium redox polymers [29]. Although these approaches demonstrated much enhanced performance of thylakoid-decorated anodes, they rely on electron transport through the secondary materials to main electrodes. 

On the other hand, there have been a relatively small number of previous works that focused on extraction of PEs from the direct contact between thylakoids and metal electrodes. Photocurrents of 100 nA/cm^2^ were measured from thylakoid-deposited indium tin oxide (ITO) electrodes without a redox mediator [30]. In another work [27], thylakoid membranes were deposited on carbon paper and stabilized by forming a thin layer of silicon oxide. From this system, a light-triggered photocurrent of ca. 150 nA/cm^2^ was observed. In this work, as shown in Figure 6a, thylakoid-deposited flat Au electrodes collected ca. 120 nA/cm^2^, which is comparable to the photocurrents from the above-mentioned works. As predicted from our hypothesis, use of thylakoid-deposited MP electrodes resulted in enhanced photocurrents of ca. 280 nA/cm^2^. This is strongly related to the increase in the ECSA of MP electrodes compared to the flat electrodes. Although MP electrodes with A/R of 1.5 have been tested in this work, the further increase of the A/R of MP electrodes may further enhance the magnitude of photosynthetic currents without sacrificing biocompatibility, stability, and voltages.

In the second cycle of light on/off for the measurement of photosynthetic currents, the photosynthetic currents from thylakoid-deposited MP electrodes started decreasing (Figure 6b). This is likely due to the presence of hydrogen peroxide that is generated during photosynthesis. Hydrogen peroxide acts as an oxidant that can damage the photosynthetic apparatus in the photosynthetic electron transport (PET) chains. Although the chloroplast has a self-eliminating system for hydrogen peroxide, isolated thylakoids lack this self-eliminating function [31] and continuous generation of hydrogen peroxide damages photosynthetic functions of the isolated thylakoids. To minimize such performance degradation by hydrogen peroxide, use of catalase, which can oxidize hydrogen peroxide into water and electrons, was proposed and stable, non-degrading performance of a thylakoid-deposited electrode was demonstrated [27,32].

There is a limited number of previous works of PFCs based on direct electron transfer. Most previous PFCs used mediator molecules to enhance the performance (indirect electron transfer). In this study, PFCs based on the MP electrode without any mediator, generated a maximum current density of about 1 μA/cm^2^ with OCV of 407 mV, which is slightly lower than 1.5–3 μA/cm^2^ with OCV of 470–650 mV measured from other systems based on carbon paper electrodes [27,33]. However, as discussed above, the anode current densities from the MP electrodes are similar to, or greater than, the values from other systems. Thus, the lower current density and OCV of the PFCs based on the MP electrodes are likely to be due to the less-optimized cathodes and cell configuration. In addition, the carbon paper electrodes of the other systems were decorated with quantum dots or stabilizing enzymes, such as catalase for enhanced performance, while the PFC in this study did not employ any secondary material. There has been no report of durability of PFCs yet, other than monitoring of PE currents for different illumination cycles as discussed above. Although a similar trend to the PE currents is expected, durability of full PFCs based on MP electrodes needs to be investigated in the near future. 

## 5. Conclusions

To maximize direct electrical contact between thylakoids and the electrodes, micro-pillar array electrodes were designed and fabricated based on the size of thylakoids. Metal-assisted chemical etching was employed to fabricate the micro-pillar array electrodes with different aspect ratios. The magnitudes of photosynthetic currents depended on the aspect ratio of the micro-pillar electrodes, which is also directly proportional to the electrochemical surface area. Without use of a mediator, photosynthetic fuel cell based on the micro-pillar electrodes were configured and maximum power output of 64 nW/cm^2^ was achieved with an OCV of 407 mV and a maximum current of about 1 mA/cm^2^.

## Figures and Tables

**Table 1:** Electrolyte resistance (*R_E_*), charge transfer resistance (*R_CT_*), internal resistance (*R_INT_*) and double layer capacitance (*C_DL_*) of flat and MP electrodes without thylakoids.

	*R_E_* (Ω)	*R_CT_* (Ω)	*R_INT_* (Ω)	*C_DL_* (F)
Flat electrode	10.3	165.8	176.1	1.32 × 10^−9^
MP electrode	4.0	109.9	113.9	1.36 × 10^−9^

**Table 2:** Electrolyte resistance (*R_E_*), charge transfer resistance (*R_CT_*), and internal resistance (*R_INT_*) of flat electrodes with different conditions.

	*R_E_* (Ω)	*R_CT_* (Ω)	*R_INT_* (Ω)
No thylakoid	10.2	165.8	176.1
With thylakoid (light off)	9.5	117	126.5
With thylakoid (light on)	9.0	112.7	121.7

**Table 3:** Comparison of the amounts of photosynthetic electrons from three different electrodes.

	Surface Area Ratio	Charge Generated (1st Cycle)	Charge Generated (2nd Cycle)
Flat	100%	6443 C	3877 C
MACE 1	133%	8014 C	5136 C
MACE 2	236%	15,545 C	8609 C

## References

[1] Mohan S.V., Velvizhi G., Modestra J.A., Srikanth S. (2014). Microbial fuel cell: Critical factors regulating bio-catalyzed electrochemical process and recent advancements. Renew. Sustain. Energy Rev..

[2] Sekar N., Ramasamy R.P. (2015). Recent advances in photosynthetic energy conversion. J. Photochem. Photobiol. C.

[3] Terasaki N., Iwai M., Yamamoto N., Hiraga T., Yamada S., Inoue Y. (2008). Photocurrent generation properties of Histag-photosystem II immobilized on nanostructured gold electrode. Thin Solid Films.

[4] Yehezkeli O., Tel-Vered R., Wasserman J., Trifonov A., Michaeli D., Nechushtai R., Willner I. (2012). Integrated photosystem II-based photo-bioelectrochemical cells. Nat. Commun..

[5] Wang W., Wang H., Zhu Q., Qin W., Han G., Shen J.-R., Zong X., Li C. (2016). Spatially separated photosystem II and a silicon photoelectrochemical cell for overall water splitting: A natural–artificial photosynthetic hybrid. Angew. Chem. Int. Ed..

[6] Ciesielski P.N., Hijazi F.M., Scott A.M., Faulkner C.J., Beard L., Emmett K., Rosenthal S.J., Cliffel D., Kane Jennings G. (2010). Photosystem I—Based biohybrid photoelectrochemical cells. Bioresour. Technol..

[7] LeBlanc G., Chen G., Gizzie E.A., Jennings G.K., Cliffel D.E. (2012). Enhanced photocurrents of photosystem I films on p-doped silicon. Adv. Mater..

[8] Gizzie E.A., Niezgoda J.S., Robinson M.T., Harris A.G., Jennings G.K., Rosenthal S.J., Cliffel D.E. (2015). Photosystem I-polyaniline/TiO_2_ solid-state solar cells: Simple devices for biohybrid solar energy conversion. Energy Environ. Sci..

[9] Ryu W., Bai S.J., Park J.S., Huang Z.B., Moseley J., Fabian T., Fasching R.J., Grossman A.R., Prinz F.B. (2010). Direct extraction of photosynthetic electrons from single algal cells by nanoprobing system. Nano Lett..

[10] Kim L.H., Kim Y.J., Hong H., Yang D., Han M., Yoo G., Song H.W., Chae Y., Pyun J.C., Grossman A.R. (2016). Patterned nanowire electrode array for direct extraction of photosynthetic electrons from multiple living algal cells. Adv. Funct. Mater..

[11] Calkins J.O., Umasankar Y., O’Neill H., Ramasamy R.P. (2013). High photo-electrochemical activity of thylakoid–carbon nanotube composites for photosynthetic energy conversion. Energy Environ. Sci..

[12] Sekar N., Umasankar Y., Ramasamy R.P. (2014). Photocurrent generation by immobilized cyanobacteria via direct electron transport in photo-bioelectrochemical cells. Phys. Chem. Chem. Phys..

[13] Liu S., Zhang P.-H., Qiu Y., Zhang J., Yu D.-Y. (2012). The photoelectrical properties of thylakoid membrane from two plants fabricated on Nano-ZnO. Mar. Sci..

[14] Yang N., Zhang Y., Halpert J.E., Zhai J., Wang D., Jiang L. (2012). Granum-like stacking structures with TiO_2_–graphene nanosheets for improving photo-electric conversion. Small.

[15] Pankratova G., Pankratov D., Di Bari C., Goñi-Urtiaga A., Toscano M.D., Chi Q., Pita M., Gorton L., De Lacey A.L. (2018). 3D graphene matrix supported and thylakoid membranes based high-performance bioelectrochemical solar cell. ACS Appl. Energy Mater..

[16] Carpentier R. (2004). Photosynthesis Research Protocols.

[17] Arnon D.I. (1949). Copper enzymes in isolated chloroplasts. Polyphenoloxidase in beta vulgaris. Plant Physiol..

[18] Granick S., Porter K. (1947). The structure of the spinach chloroplast as interpreted with the electron microscope. Am. J. Bot..

[19] Dekker J.P., Boekema E.J. (2005). Supramolecular organization of thylakoid membrane proteins in green plants. Biochim. Biophys. Acta.

[20] Austin J.R., Staehelin L.A. (2011). Three-dimensional architecture of grana and stroma thylakoids of higher plants as determined by electron tomography. Plant Physiol..

[21] Bard A., Faulkner L. (2001). Electrochemical Methods: Fundamentals and Applications.

[22] Sekar N., Ramasamy R.P. (2013). Electrochemical impedance spectroscopy for microbial fuel cell characterization. J. Microb. Biochem. Technol..

[23] O’hayre R., Cha S.-W., Prinz F.B., Colella W. (2016). Fuel Cell Fundamentals.

[24] Huang Z.P., Geyer N., Werner P., de Boor J., Gosele U. (2011). Metal-assisted chemical etching of silicon: A review. Adv. Mater..

[25] Zhang M.L., Peng K.Q., Fan X., Jie J.S., Zhang R.Q., Lee S.T., Wong N.B. (2008). Preparation of large-area uniform silicon nanowires arrays through metal-assisted chemical etching. J. Phys. Chem. C.

[26] Li X. (2012). Metal assisted chemical etching for high aspect ratio nanostructures: A review of characteristics and applications in photovoltaics. Curr. Opin. Solid State Mater. Sci..

[27] Sjoholm K.H., Rasmussen M., Minteer S.D. (2012). Bio-solar cells incorporating catalase for stabilization of thylakoid bioelectrodes during direct photoelectrocatalysis. ECS Electrochem. Lett..

[28] Lee J., Im J., Kim S. (2016). Mediatorless solar energy conversion by covalently bonded thylakoid monolayer on the glassy carbon electrode. Bioelectrochemistry.

[29] Hamidi H., Hasan K., Emek S.C., Dilgin Y., Akerlund H.E., Albertsson P.A., Leech D., Gorton L. (2015). Photocurrent generation from thylakoid membranes on osmium-redox-polymer-modified electrodes. Chemsuschem.

[30] Dewi H.A., Meng F.B., Sana B., Guo C.X., Norling B., Chen X.D., Lim S.R. (2014). Investigation of electron transfer from isolated spinach thylakoids to indium tin oxide. RSC Adv..

[31] Asada K. (2006). Production and scavenging of reactive oxygen species in chloroplasts and their functions. Plant Physiol..

[32] Yeung C.H., de Geyter C., de Geyter M., Nieschlag E. (1996). Production of reactive oxygen species by and hydrogen peroxide scavenging activity of spermatozoa in an IVF program. J. Assist. Reprod. Genet..

[33] Rasmussen M., Wingersky A., Minteer S.D. (2014). Improved performance of a thylakoid bio-solar cell by incorporation of carbon quantum dots. ECS Electrochem. Lett..

